# Improving multimorbidity measurement using individualized disease-specific quality of life impact assessments: predictive validity of a new comorbidity index

**DOI:** 10.1186/s12955-022-02016-7

**Published:** 2022-07-12

**Authors:** Mindy L. McEntee, Barbara Gandek, John E. Ware

**Affiliations:** 1 College of Health Solutions, Arizona State University, 500 N. 3rd Street, Phoenix, AZ 85004-0698 USA; 2 University of Massachusetts Medical School, Worchester, MA USA; 3 John Ware Research Group, Inc., Watertown, MA USA

**Keywords:** Disease-specific outcomes, Health-related quality of life, Comorbid conditions, Quality of life disease impact scale (QDIS), Charlson comorbidity index (CCI)

## Abstract

**Background:**

Interpretation of health-related quality of life (QOL) outcomes requires improved methods to control for the effects of multiple chronic conditions (MCC). This study systematically compared legacy and improved method effects of aggregating MCC on the accuracy of predictions of QOL outcomes.

**Methods:**

Online surveys administered generic physical (PCS) and mental (MCS) QOL outcome measures, the Charlson Comorbidity Index (CCI), an expanded chronic condition checklist (CCC), and individualized QOL Disease-specific Impact Scale (QDIS) ratings in a developmental sample (N = 5490) of US adults. Controlling for sociodemographic variables, regression models compared 12- and 35-condition checklists, mortality vs. population QOL-weighting, and population vs. individualized QOL weighting methods. Analyses were cross-validated in an independent sample (N = 1220) representing the adult general population. Models compared estimates of variance explained (adjusted R^2^) and model fit (AIC) for generic PCS and MCS across aggregation methods at baseline and nine-month follow-up.

**Results:**

In comparison with sociodemographic-only regression models (MCS R^2^ = 0.08, PCS = 0.09) and Charlson CCI models (MCS R^2^ = 0.12, PCS = 0.16), increased variance was accounted for using the 35-item CCC (MCS R^2^ = 0.22, PCS = 0.31), population MCS/PCS QOL weighting (R^2^ = 0.31–0.38, respectively) and *individualized* QDIS weighting (R^2^ = 0.33 & 0.42). Model R^2^ and fit were replicated upon cross-validation.

**Conclusions:**

Physical and mental outcomes were more accurately predicted using an expanded MCC checklist, population QOL rather than mortality CCI weighting, and *individualized* rather than population QOL weighting for each reported condition. The 3-min combination of CCC and QDIS ratings (QDIS-MCC) warrant further testing for purposes of predicting and interpreting QOL outcomes affected by MCC.

**Supplementary Information:**

The online version contains supplementary material available at 10.1186/s12955-022-02016-7.

## Introduction

Over a quarter of US adults (27.2%) have been diagnosed with multiple chronic conditions (MCC) that adversely impact health status, functioning, or health-related quality of life (QOL), with greater prevalence among women, non-Hispanic white adults, those over age 65, and those living in rural areas [1]. One-third have three or more. Because MCC are predictive of mortality, disability, response to treatment, health care utilization, expenditures, and declines in QOL (particularly physical QOL) [2], case mix adjustments for differences in MCC have become essential in health outcomes and effectiveness research.[3–9]. As noted with the first clinical definition of comorbidity[10] and other clinimetric principles used in developing the first comorbidity indices[11], MCC data can also enhance the staging of individual patient complexity, aid in treatment planning, and improve application of quality of care guidelines [12–14]. Therefore, more accurate estimates of MCC impact have potential for improving adjustments for case mix differences in comparative effectiveness research and provider outcome comparisons [12–17], and play a critical role in improving patient care by identifying individuals most likely to benefit from specific treatments/services [18, 19].

QOL is a patient-reported outcome (PRO) of particular importance in the movement toward patient-centered care and shared decision-making. While the relevance of QOL status and outcomes is straightforward, their clinical utility is predicated on the use of reliable and validated measurement that is sensitive to change[20] and satisfies other clinimetric principles [11, 21, 22]. QOL assessments fall into two categories—generic or disease-specific measures. One major difference between these measurement categories is whether QOL is attributed to a specific disease or diagnosis [23, 24]. Generic QOL measures have the advantage of enabling comparisons of disease burden across MCC, while disease-specific QOL measures provide greater responsiveness to a specific condition [23]. As hypothesized decades ago[23], disease-specific QOL measures that summarize the impact attributed to one disease have been shown to be a substantial improvement in clinical usefulness compared to generic measures of the same QOL domains [25–27].

At the heart of all disease-specific QOL impact attributions is the assumption that patients can validly parse the impact of any one of their conditions in the presence of MCC, a common situation in the interpretation of outcomes. This assumption has been supported by comparisons of the validity of specific and generic measurement methods within a given clinical condition [28, 29], including a large US chronically ill population study results showing significant convergent and discriminant validity across 90% of 924 tests of MCC within nine pre-identified disease conditions. Specifically, tests comparing correlations among different methods (e.g., clinical markers and disease-specific QOL ratings) measuring the same condition (i.e., convergent validity) were substantial in magnitude and significantly higher than correlations between comorbid diseases measured using the same method (i.e., discriminant validity) [27]. Notably, previous evaluations of validity for a specific condition have almost always been limited to convergent evidence, i.e., that different methods measuring the same condition reach substantial agreement.

Thus far, the assessment of MCC impact has been hindered by a proliferation of diverse measures and a dearth of studies addressing the practical implications of differences across MCC assessment methods. For example, legacy methods for aggregating MCC have been based on simple condition count, which ignores differences between conditions and assumes that the impact of each condition is the same for all who have it [2, 9, 17, 30–33]. Methods addressing those differences have weighted conditions on a population level using criteria such as mortality [2, 34] or health care utilization [24]. Among the first MCC measures to recognize the importance of both the number of conditions and the differences in their impact, the Charlson Comorbidity Index (CCI) [34] has been the most frequently and extensively studied [2]. The Elixhauser alternative expands the list of conditions [35]. While shown to be useful for some case mix adjustment purposes, these indexes have been criticized for their reliance on *mortality* weighting, omission of prevalent and morbid conditions, and assignment of the same population weight to everyone with a given condition. Emphasis on generic QOL outcomes monitoring and evidence regarding the substantial impact of prevalent and morbid conditions (e.g. osteoarthritis, back problems, depression) omitted from the CCI are known to affect the generic physical, psychological, and social QOL domains [2].

In response, QOL-based patient reported outcome (PRO) monitoring research has spawned several advances in MCC assessment methods, including approaches using (1) models that standardize disease-specific QOL impact scoring across different conditions, (2) a summary disease-specific impact score aggregating across QOL domains (i.e., simplified 1-factor scoring), (3) IRT-based calibrations of single and multi-item measure scoring across diseases [24, 25, 36, 37], and (4) items proven to discriminate QOL impact for a given condition in the presence of other comorbid conditions. Scoring can distinguish between *comorbidity, or* impact in the context of an “index” condition in tertiary and secondary care settings, and *multimorbidity (*total QOL impact in primary care and other generalist settings). [38, 39]

Until recently, a lack of standardization of QOL content and scoring across *disease-specific* QOL measures has impeded meaningful comparisons across conditions and aggregation of total MCC QOL impact. Prior work has addressed this issue with disease-specific QOL assessments reflecting the richness of widely-used generic QOL surveys and standardized across diseases with items differing only in terms of disease-specific attributions for QOL impact [25, 40].

The practical implications of a *single factor* disease-specific measurement model leaving very little disease-specific QOL shared item variance unexplained[36, 37] is that it enables a summary score for each condition with minimal loss of information and reduced respondent burden in comparison with multiple scores for each condition [24, 37]. Other practical implications include greater measurement efficiency, standardized comparisons of disease-specific QOL burden, improved aggregation of QOL impact across MCC, and adaptive *disease-specific* QOL assessments [24, 25, 27, 41]. Results showing high *single item* correlations with *individualized* disease-specific QOL item bank total scores enabled further reduction in respondent burden for estimating MCC impact for each individual [24, 26, 27, 36, 37]. Finally, measures such as the QDIS-MCC used in the current study reflect an advance, with rare exceptions, in both convergent and discriminant validity for priority MCC [27, 28, 42].

This paper examines whether an expanded condition checklist, population QOL-weighting (in contrast to mortality weighting), and use of each patient’s own QOL impact rating (individualized ratings in contrast to population weights for their MCC) improve predictions of generic QOL outcomes. To date, these methods have only been tested on small [43–45] or age-restricted samples.[46]. This is the first study to systematically compare legacy and improved methods for aggregating the impact of MCC with the goal of better understanding their effects on the accuracy of predictions of generic physical and mental QOL status and outcomes.

## Materials and methods

Data was obtained from US adults completing internet surveys as part of the Computerized Adaptive Assessment of Disease Impact (DICAT) study, which sought to develop and evaluate standardized disease-specific QOL measures to aggregate impact across MCC. This study was approved by the New England Institutional Review Board; details regarding sampling and data collection methods are published elsewhere [24]. Briefly, independent samples from an ongoing GfK research panel of approximately 50,000 adults were drawn in three waves at different times in 2011, with email and automated telephone reminders sent to non-responders. Cross-sectional data was collected from new participants in all waves; those recruited in the first two waves completed longitudinal surveys at six- and nine-month follow-up.

### Developmental sample

The first analytic sample consisted of adults previously diagnosed with any of nine chronic conditions (pre-ID group) plus a random subset of general population respondents (N = 350) who endorsed zero chronic condition checklist items to enable a comparison group (i.e., the intercept of the regression models). Pre-ID conditions were categorized in five groups: arthritis (osteoarthritis [OA], rheumatoid [RA]), chronic kidney disease (CKD), cardiovascular disease (angina, myocardial infarction [MI] in past year, congestive heart failure [CHF]), diabetes, and respiratory disease (asthma, chronic obstructive pulmonary disease [COPD]). Of the 9160 pre-ID panelists invited to participate, 6828 opened the informed consent screen (74.5%), 5585 consented, and 5418 completed surveys (survey completion rate 97.0%). Panelists were sampled to achieve at least 1000 respondents within three priority disease groups, with smaller targets for less prevalent diagnoses (CKD, cardiovascular). Conditions were confirmed at the start of the internet survey. All MCC aggregation models were developed and compared with this sample.

### Cross-validation sample

The second analytic sample represented the US general adult population, including those with and without chronic conditions in their naturally occurring proportions. Of the 10,128 panelists sent invitations, 6433 (63.5%) opened the informed consent screen, 5332 consented, and 5173 completed surveys (survey completion rate 97.0%). As noted above, 350 randomly selected participants reporting no chronic conditions were excluded from cross-validation analyses due to their inclusion in the developmental sample for comparison purposes. Remaining general population data were analyzed to cross-validate all cross-sectional and longitudinal models.

### Measures & protocol

Survey items (modules) varied by design within samples and recruitment waves [24]. Random assignment to survey protocols within each wave enabled comparisons of longer and shorter survey modules (i.e., full length or fast track) to test the effects of differences in respondent burden (median total time limited to ≤ 25 min). For all protocols, modules were administered in the following order: generic QOL measures, chronic condition checklist, QOL disease-specific (QDIS) items, and legacy disease-specific measures (developmental sample only). Internet-based electronic data collection (EDC) allowed data quality to be monitored in real time. EDC date and timestamp estimates were used to estimate respondent burden for all forms. Completeness of survey responses was not an issue: QDIS items had missing data rates of 0.6–1.4%.

#### Generic QOL measures

All panelists completed the SF-8™ Health Survey [47], used to estimate favorably-scored generic physical (PCS) and mental (MCS) component summaries at all time points [48]. A random 40% subsample also completed the SF-36® Health Survey [49] to replicate PCS and MCS results using more reliable 36-item estimates. All PCS and MCS scores were normed to have mean = 50 and SD = 10 using standardized developer scoring; SF-8 and SF-36 estimates were highly correlated (0.899 and 0.868, respectively), as in previous studies [47, 49, 50].

#### MCC presence and disease-specific impact measures

Disease-specific QOL measures included the Charlson Comorbidity Index (CCI) [34] and QOL-weighted Disease Impact Scale for Multiple Chronic Conditions (QDIS-MCC) using responses to a 35-item chronic condition checklist based on the National Health Interview Survey (NHIS) [51], Medicare Health Outcomes Survey [52], and US department of Health and Human Services (HHS) priorities [42] with common self-report instructions. Specifically, the checklist asks if a doctor or other health professional had ever told the respondent they had or currently have any of the listed conditions. For each condition endorsed, the CCI applied population mortality weights and the QDIS-MCC administered a global QOL Disease-specific Impact Scale (QDIS) item asking “In the past 4 weeks, how much did your < condition > limit your everyday activities or your quality of life?” with categorical response choices ranging from Not at all to Extremely, scored to indicate greater impairment in QOL [24, 37].

As illustrated in the appended paper–pencil form (see Additional file 1), the current study administered items for each condition on the left side using a skip pattern, presenting a global impact item for each endorsed condition. Individualized QDIS-MCC scores were calculated for each respondent by summing global impact scores across all endorsed conditions. Thus, both CCI and QDIS-MCC total scores reflect the number of conditions reported and their impact on each individual. They differ in multiple respects, as documented in Table 3. The global QDIS item used to estimate disease-specific QOL impact in this study has been shown to be consistently correlated highly (r > 0.80) with the same-disease QDIS item bank score and significantly lower (r < 0.40) with item bank scores for comorbid conditions [24, 37]. As noted above, tests of convergent validity across multiple methods (clinical, QOL) for measuring each condition and discriminant validity across conditions for the global QDIS item using multitrait-multimethod analyses substantially support its ability to distinguish QOL impact for one condition (e.g. OA) in the presence of comorbid conditions (e.g., asthma and diabetes), with rare exceptions [27, 28].

To facilitate interpretation by disease condition and other demographic groups, QDIS scale scores were normed in 2010 in representative samples of the U.S. chronically ill population and transformed to have mean = 50, SD = 10) [25]. Scores above and below 50 are above and below the chronically ill population average across all conditions and can be evaluated easily in SD units. The same linear T-score transformation has been applied to the aggregate individualized QDIS-weighted MCC impact score in the US chronically ill population cross validation sample (Table 3).

#### Demographic covariates

Baseline regression models controlled for 16 categories including age (18–34, 44–54, 45–54, 55–64, 65–74, 75 +), gender, race/ethnicity (white non-Hispanic, black non-Hispanic, Hispanic, other non-Hispanic) and education (less than high school graduate, high school graduate, post-high school education, college graduate or higher).

### Analytic plan

#### Aim 1: systematic evaluation of different methods of aggregating disease-specific QOL impact in estimating generic patient-reported outcomes

As in previous studies [53–55], ordinary least squares regression models, controlling for respondent characteristics, predicted PCS and MCS using both cross-sectional and longitudinal data. Systematic comparisons examined multiple methods of MCC aggregation, including condition counts, population-weighted scores, and an extension of these models that included each individual’s responses to global QDIS items (individualized score; see Table 1). This allowed for a number of model comparisons between legacy and newer aggregation methods, including:

- Addition of simple count of 35 (#1) or 12 (Charlson) conditions (#1C) to sociodemographic-only base model (#0)
- Simple counts (#1) vs. population weights (#2-M and #2-P)
- Population QOL or mortality weights (#2 and #2C) vs. individualized QDIS ratings (#3 and #3C)

**Table 1:** Summary and description of QDIS-MCC, legacy, and Charlson impact estimation methods and models compared

Model & MCC Estimation Method		Base	Count	Pop. weights	Ind. QDIS items	Description of MCC Impact Score (MCCIS)
*QDIS-MCC Models*						
0	Base model—sociodemographics only	X				
1	Simple chronic conditions count	X	X			Count of all reported conditions on expanded checklist
2–M/P	Population-weighted MCS/PCS score	X		X		Separate regressions for MCS & PCS on 35 (0/1) conditions in developmental sample; weighted sum of all reported conditions using corresponding MCS/PCS population weights
3	Individualized QDIS-weighted MCC score	X			X	Separate regressions for MCS/PCS, individualized by adding global QDIS ratings for all reported conditions (for each participant)
*Charlson Models*						
1C	Charlson 12 count	X	X			Sum of all reported Charlson conditions
2C–M/P	MCS-/PCS- weighted Charlson score	X		X		Separate regressions for MCS & PCS on 12 (0/1) Charlson conditions in developmental sample; weighted sum of all reported Charlson conditions using corresponding MCS or PCS population weights
3C	Individualized QDIS-weighted Charlson	X			X	Separate regressions for MCS & PCS; individualized by adding global QDIS ratings for all reported Charlson conditions (for each participant)
4C	Mortality-weighted Charlson score	X		X		Pseudo-Charlson score for 12 conditions using 1987 published mortality rates

MCC = multiple chronic conditions, pop. = population, ind. = individualized, PCS = Short Form-8 Physical Component Score, MCS = Short Form-8 Mental Component Score

Model contrasts include:

0 vs. 1 or 1C: Sociodemographic-only base model (0) versus expanded 35-condition checklist (1) or Charlson 12 condition count (1C)

1 vs. 2-M/P: Expanded checklist condition count (1) versus population QOL weights for expanded checklist conditions (2-M/2-P)

1C vs. 2C-M/P: Charlson 12 count (1C) versus population QOL weights for Charlson 12 conditions (2C-M/2C-P)

2-M/P vs. 3: Population-weighted QOL (2-M/2-P) versus individualized QDIS ratings for expanded condition checklist (3)

2C-M/P vs. 3C: Test of population-weighted QOL versus individualized QDIS ratings for Charlson 12 conditions

4C vs. 3C: Test of mortality-weighted Charlson versus individualized QDIS ratings for Charlson 12 conditions

Population weights were derived independently for PCS and MCS in the developmental sample using regressions of dummy variables (yes = 1/no = 0) for each condition. These weights, which were applied to the 35 yes/no condition indicators (Model #2-M, 2-P) reflect the number of MCC and the average population impact of each disease on MCS and PCS controlling for all other conditions. For individualized models (#3 and #3C, respectively, for 35 and 12 conditions) QDIS ratings were summed for conditions reported. For all models, 16 dummy variables controlled for categories of four sociodemographic characteristics: age, gender, race/ethnicity, and education. Model methods were ordered on the basis of their hypothesized incremental validity [56, 57], a type of validity used to empirically test how much a new method will improve predictive ability beyond what is provided by an existing method. Cross-sectional analyses combined participant baseline data obtained at study entry across waves. To test how well current MCC estimates predicted future health, longitudinal models used general population baseline data (including weights) to predict PCS and MCS at nine-month follow-up.

To standardize comparisons between mortality weighted and QOL weighted model effects, both were limited to the 12 conditions common to the Charlson (CCI) [34] and 35-condition checklist, as identified in Table 4. Some noteworthy constraints applied to all CCI models. As with other studies reliant on self-reported CCI data [58], the 35-condition checklist did not include Charlson peripheral vascular disease or dementia and definitions for common conditions sometimes varied. Hemiplegia was counted if a stroke and limitations in the use of an arm or leg (missing, paralyzed, or weakness) was reported. Data was not available to distinguish between mild and severe CCI levels for diabetes, liver disease, and cancer. All were conservatively scored at the lower CCI level, consistent with other studies [59]. Kidney disease was based on participant report of serum creatinine and converted to the estimated glomerular filtration rate; [60] participants with eGFR < 60 were classified as having kidney disease. Due to DICAT study design, serum creatinine was only available for the participants in the developmental sample pre-identified with CKD; kidney disease was not included as a condition in cross-validation analyses.

Aggregations of CCI conditions paralleled methods used with the 35-condition checklist, including condition counts, population-weights, and individualized scores. CCI model tests and results were ordered to correspond with analogous QDIS-MCC models. A fourth model utilized CCI published mortality weights. Population weights (Model #2C-M, 2C-P) were derived and comparisons made using the same methods described above for Model #2.

Adjusted R^2^ was estimated for each regression model and was the primary outcome of interest. Akaike information criterion (AIC) [61] was used for objective interpretation and model comparison; smaller AICs indicate more parsimonious models. Model comparisons focused on the difference in AIC (delta) between a referent model and an alternative nested model hypothesized to be an improvement. As recommended, the absolute value of AIC estimates were ignored as were deltas < 2 [62, 63]. Deltas > 10 were accepted as sufficient evidence that models with larger AICs performed worse. Analyses were conducted in Stata (Stata Corp, College Station, TX). AIC has previously been reported in studies comparing performance of comorbidity indices [64–66].

**Fig. 1:**
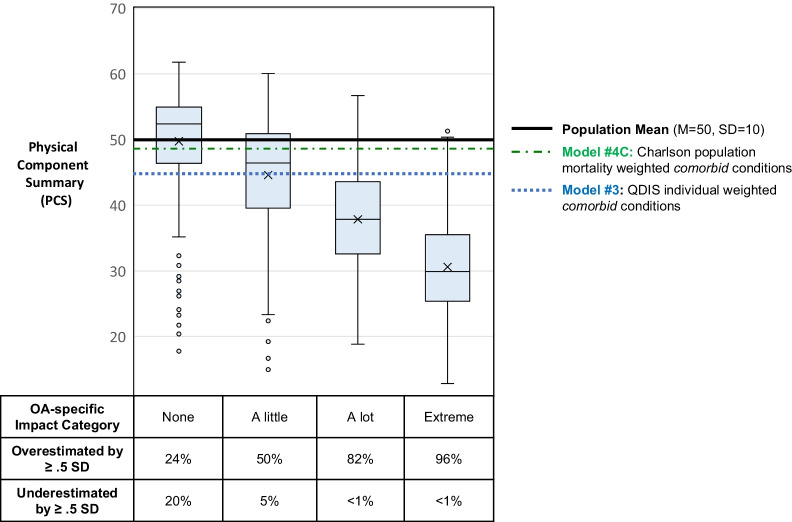
Effects of MCC impact adjustment methods on PCS outcome predictions by OA-specific QOL impact

To estimate and illustrate the practical implications of differences in magnitude of case-mix adjustments, Fig. 1 compares adjusted PCS scores for participants with OA in the developmental sample (N = 1135) at baseline. These OA-sample-only analyses controlled for baseline sociodemographic variables as in analyses for the total sample as described above (also listed in Table 5 footnotes). Model #2 applied the OA-specific PCS population adjustment to everyone; Model #4C applied the CCI mortality adjustment to everyone; whereas Model #3 applied the individualized QDIS global item adjustment distinguishing groups differing in impact attributed to OA (with A lot and Extreme categories combined). Accordingly, population-weighted models, which assign the same adjustment to everyone with OA, appear as higher or lower horizontal lines across levels of OA impact in the Figure. In contrast, Model #3 individualized QDIS-OA adjustments are graphically displayed as four box plots, each indicating the median, interquartile, and total range of PCS outcomes adjusted for QDIS individualized impact attributions.

#### Aim 2: cross-validation of MCC impact model comparisons

All cross-sectional and longitudinal models above were cross-validated independently (cross-validation sample, N = 3416) to examine the generalizability of results. Adjusted R^2^ and AIC [61] were used for interpretation of model predictive validity and parsimony using the same methods for as described above for Aim 1.

## Results

### Samples and measures

Demographic characteristics for the developmental and cross-validation samples, as summarized in Table 2, were similar except that the developmental sample tended to be older, on average, and less often unemployed. Prevalence rates for each condition ranged from 0.5 to 11.3% for the 12 Charlson (CCI) conditions and from 0.5 to 8.4% for the 35 checklist conditions in the more representative cross-validation sample. With one exception (HIV or AIDS), prevalence rates were consistently much higher for the developmental sample as expected given the oversampling of pre-ID conditions. The percentage of respondents reporting one or more conditions was much lower (28.6%) for the 12-condition CCI in comparison to the 35-condition checklist (76.8%). MCC counts ranged from 0 to 31; distributions were highly skewed for the 35-condition checklist, with 35% requiring individualized QDIS impact item administrations for four or more conditions (Model #3). Accordingly, respondent burden varied substantially (range = 0–65 items; interquartile range = 1–14 items; median = 7 items), with EDC-monitored administration times for the combined chronic condition checklist and single global QDIS impact item for each reported condition ranging from 2 to 3 min for most respondents.

**Table 2:** Characteristics of developmental and cross-validation samples

	Developmental Sample (N = 5418)	Cross-Validation Sample (N = 3949)		Developmental Sample (N = 5418)	Cross-Validation Sample (N = 3949)
Age			Condition (%)		
Mean (SD)	59.5 (13.7)	48.3 (16.5)	Angina	8.0	2.1
Range	18–7	18–94	Myocardial infarction^c^	2.3	0.7
Male (%)	42.8	49.5	Congestive heart failure^c^	8.8	1.7
Race/Ethnicity (%)			Diabetes^c^	41.1	11.2
White non-Hispanic	79.9	77.8	Asthma^c^	31.3	11.3
Black non-Hispanic	7.9	8.5	COPD^c^	11.7	3.4
Hispanic	5.9	8.0	Kidney disease^c^	7.2	1.8
Other non-Hispanic	6.3	5.7	Osteoarthritis	38.8	11.1
Education (%)			Rheumatoid arthritis^c^	16.7	5.1
< HS graduate	3.3	6.7	Allergies, chronic	33.8	19.2
High school graduate	18.4	27.4	Allergies, seasonal	50.4	36.6
Some college	38.0	31.9	Anemia	16.6	9.2
College graduate	40.3	34.0	Cancer (non-skin) ^c^	11.6	6.1
Income (%)			Chronic back problems	33.9	17.9
< $20,000	12.5	14.2	Chronic fatigue syndrome	4.3	2.2
$20,000–39,999	21.6	21.1	Depression	19.5	12.1
$40,000–99,999	48.6	41.9	Dermatitis/skin conditions	14.9	9.4
$100,000 +	17.3	22.8	Enlarged prostate (BPH)	11.2	5.3
Employment Status (%)			Erectile dysfunction	12.6	5.4
Employed	38.4	54.9	Fibromyalgia	7.2	2.6
Unemployed	4.3	7.2	Hearing, trouble	20.7	10.7
Retired due to age	32.5	16.2	HIV or AIDS^c^	0.5	0.5
Disabled^a^	14.3	6.7	Hypertension	58.4	30.9
Other^b^	9.8	14.1	Hypothyroidism	16.9	8.5
Missing	0.7	0.9	Irritable bowel syndrome	12.8	7.3
			Joint problems, foot/ankle	20.2	9.9
Generic QOL Mean (SD)			Joint problems, hip/knee	40.9	19.9
SF-8 MCS	49.3 (9.9)	50.5 (9.4)	Limb, limitations in use	12.8	5.8
			Liver Disease^c^	3.1	1.9
SF-8 PCS	44.2 (10.5)	50.0 (9.0)	Migraine headaches	18.7	13.1
			Obesity	35.0	17.2
			Osteoporosis	11.4	5.1
			Stroke^c^	5.2	2.1
			Ulcer/stomach disease^c^	13.8	7.8
			Vision, trouble	15.0	9.2

^a^Retired due to disability. ^b^Homemaker, student, other. Joint problems and limb limitations were not counted as separate conditions if the participant reported osteoarthritis or rheumatoid arthritis. ^c^Condition used in estimating Charlson CI

Descriptive statistics for all MCC aggregation methods across models and generic QOL outcomes in the developmental sample are summarized in Table 3. The highly variable individualized MCC scores (range = 0–91, mean = 10.94) used in Model #3 predictions reflect the combined effects of reporting more conditions (range = 0–31, mean = 5.78) as well as differences in their QOL impact. Mean PCS for the developmental sample was approximately ½ SD lower for the developmental sample, as expected for a sample over-representing primarily physical conditions.

**Table 3:** Summary information for MCC methods, models, and generic outcomes in cross-sectional developmental sample (N = 5,490)

Models & Outcome Variables	# of CC	Simple Count	Pop. Weight MCS	Pop. Weight PCS	Ind. QDIS Weight	Pop. Weight Mortality	Mean	SD	Range*
*QDIS-MCC Methods*									
1: Simple chronic conditions count	35	X					5.78	3.49	0–31
2–M: Population-weighted MCS score	35		X				47.81	5.01	20.49–53.23
2–P: Population-weighted PCS score	35			X			44.65	6.49	3.43–53.87
3: Individualized QDIS-weighted MCC score	35				X		10.94	9.53	0–91
4: Individualized OA comorbid CC^a^ score	35				X		16.74	12.6	0–102
*Charlson Index Methods*									
1C: Charlson 12 count	12	X					1.37	1.09	0–10
2C–M: MCS-weighted Charlson score	12		X				46.57	2.57	26.18–49.40
2C–P: PCS-weighted Charlson score	12			X			44.23	3.66	18.63–47.91
3C: Individualized QDIS-weighted Charlson	12				X		2.32	2.61	0–26
4C: Mortality-weighted Charlson score	12					X	0.68	1.14	0–16
*Generic Outcomes*									
MCS							49.59	9.76	10.1–68.5
PCS							44.82	10.5	12.8–66.3

*CC*  chronic conditions, *pop.*  population, *ind.*  individualized, *PCS*  Short Form-8 Physical Component Score, *MCS*  Short Form-8 Mental Component Score.*Observed range. ^a^Displayed in Fig. 1 example limited to OA index condition (N = 1235) comorbidity impact

### Population-based PCS and MCS Weights for chronic conditions

As shown in Table 4, unique effect estimates for nearly all 35 conditions were negative in predictions of both PCS (34/35) and MCS (31/35) and significant for PCS (23/35) and MCS (17/35), indicating worse QOL. Overall, effects (weights) tended to be larger for PCS, exceeding 0.25 SD (b > 2.50) for 10 conditions (CHF, stroke, COPD, RA, OA, chronic fatigue, fibromyalgia, chronic back, limited use or arm/leg), compared to three for MCS (depression, chronic fatigue, and vision problems). With few exceptions, conditions not included in the CCI showed significant effects on one or both outcomes. As expected for respondents without chronic conditions (holdout group), the intercept estimates for Models #1–3 (all but the base model) were well above the population mean of 50 for both PCS and MCS.

**Table 4:** Estimates of unique effects of chronic conditions on MCS and PCS used in MCC weighting, developmental sample (N = 5490)

Chronic condition^a^	N	MCS			PCS		
		b	SE	t	b	SE	t
Hypertension	3185	−0.27	0.25	−1.05	−1.40	0.26	−5.40***
*Myocardial infarction*	123	−0.76	0.77	−0.99	−2.43	0.78	−3.10**
Angina	433	0.05	0.44	0.11	−1.36	0.45	−3.04**
*CHF*	477	−1.24	0.42	−2.96***	−3.67	0.43	−8.59***
*Diabetes*	2231	−0.64	0.26	−2.45*	−1.17	0.27	−4.40***
*Stroke*	285	−0.95	0.53	−1.77	−2.59	0.54	−4.78***
*Cancer*	629	−0.95	0.37	−2.60***	−0.60	0.37	−1.63
*Asthma*	1700	−0.77	0.28	−2.80**	−0.51	0.28	−1.80
*COPD*	634	−0.94	0.37	−2.52*	−4.27	0.38	11.36***
*CKD*	387	−0.14	0.46	−0.31	−1.82	0.46	−3.94***
*RA*	909	−0.97	0.32	−3.00**	−4.35	0.33	−13.26***
OA	2107	−0.24	0.28	−0.84	−4.90	0.29	−17.08***
Osteoporosis	623	−0.08	0.38	−0.20	−1.13	0.39	−2.93**
*Ulcer*	753	−0.99	0.35	−2.85**	−0.94	0.35	−2.67*
*Liver disease*	169	−0.78	0.67	−1.16	−0.88	0.68	−1.29
Irritable bowel syndrome	694	−1.57	0.36	−4.35***	−0.36	0.37	−0.98
Obesity	1900	−0.77	0.26	−2.93**	−1.76	0.26	−6.63***
*HIV or AIDS*	27	−0.17	1.63	−0.11	−0.10	1.65	−0.06
Anemia	897	−0.29	0.33	−0.91	−0.64	0.33	−1.95
Depression	1058	−7.82	0.31	−25.09***	−0.08	0.32	−0.26
Chronic fatigue syndrome	235	−3.55	0.61	−5.81***	−3.25	0.62	−5.25***
Fibromyalgia	390	−1.67	0.49	−3.40**	−3.43	0.50	−6.87***
Migraine headaches	1013	−0.58	0.31	−1.84	−0.63	0.32	−1.97*
Prostate disease	616	0.01	0.41	0.03	−0.47	0.42	−1.12
Erectile dysfunction	684	−0.87	0.40	−2.20*	−1.00	0.40	−2.48*
Hypothyroidism	919	0.18	0.32	0.57	0.00	0.32	−0.01
Allergies, chronic	1836	−0.24	0.27	−0.89	−0.10	0.27	−0.37
Allergies, seasonal	2739	0.19	0.24	0.76	0.92	0.25	3.70***
Chronic back problem	1840	−1.63	0.27	−6.17***	−4.15	0.27	−15.47***
Vision trouble	814	−2.64	0.33	−7.89***	−2.15	0.34	−6.33***
Hearing, trouble	1124	−0.43	0.30	−1.46	−0.80	0.30	−2.66***
Osteoporosis	812	−0.84	0.32	−2.59*	−0.92	0.33	−2.80**
Limited use arm/leg	697	−1.12	0.62	−1.82	−6.55	0.63	−10.42
Foot/ankle joint problem	1099	−1.74	0.55	−3.16**	−1.42	0.56	−2.54*
Hip/knee joint problem	2225	−0.15	0.42	−0.35	−2.49	0.42	−5.87***
Constant (Intercept)		51.93	0.44		53.46	0.40	

*MCS*  Short Form-8 Mental Component Score, *PCS*  Short Form-8 Physical Component Score. ^a^Conditions common to 35-item and Charlson checklists are in *italics.* ***p < 0.001; **p < 0.01; *p < 0.05. Each regression model adjusted for main effects of age, gender, education, and race/ethnicity

### Evaluations of MCC impact aggregation methods and models

Comparisons of MCC impact aggregation methods demonstrated the increased explanatory power of all methods over the sociodemographic-only base model, with all F-ratios > 38.2, all *p* < 0.0001. As shown in Table 5, cross-sectional models (#1–3) progressively improved explanatory power in a manner consistent with hypotheses, as evidenced by increases in adjusted R^2^ and decreased AIC values, the latter indicating more parsimonious models for deltas between models satisfying the > 10 criterion. Specifically, in comparison with sociodemographic-only regression models (MCS R^2^ = 0.08, PCS = 0.09) and Charlson CCI models (MCS R^2^ = 0.12, PCS = 0.16), the variance explained in PCS increased to 31% using simple MCC count (Model #1), to 39% using PCS population weighted scores for 35 conditions (Model #2-P), and to 42% using individualized weights for those conditions (Model #3). Variances explained in MCS increased to 22% with simple MCC count (Model #1), 31% using MCS population weights for 35 conditions (Model #2-M), and 33% using individualized condition weights (Model #3). In support of the generalizability of cross-sectional results, the above pattern of increased predictive validity (adjusted R^2^) and improved model parsimony (AIC) progressively across the models was replicated in analyses limited to 12 CCI conditions (see the bottom half of Table 5). Further, QOL-weighted models (#1C-3C) consistently outperformed population mortality-weighted CCI in analyses limited to 12 CCI conditions (Model #4C).

**Table 5:** Summary of variances explained and model fit by method and model, developmental sample

Model & Description		df^a^	Cross-sectional models N = 5490				Longitudinal models N = 2170			
			MCS		PCS		MCS		PCS	
			Adj R^2^	AIC	Adj R^2^	AIC	Adj R^2^	AIC	Adj R^2^	AIC
*QDIS-MCC Models*										
0	Base model—sociodemographic variables only^b^	12	0.08	40,181	0.09	40,905	0.06	15,970	0.09	16,145
1	Simple chronic conditions count	13	0.22	39,221	0.31	39,400	0.17	15,691	0.26	15,675
2–M/P	Population-weighted MCS/PCS score^c^	47	0.31	38,560	0.39	38,656	0.25	15,479	0.34	15,433
3^d^	Individualized QDIS-weighted MCC score	13	0.33	38,435	0.42	38,460	0.26	15,438	0.36	15,383
*Charlson Models*										
1C	Charlson 12 count	13	0.13	39,858	0.18	40,340	0.10	15,884	0.15	15,987
2C–M/P	MCS-/PCS-weighted Charlson score	24	0.13	39,817	0.19	40,237	0.10	15,861	0.16	15,970
3C	Individualized QDIS-weighted Charlson score	13	0.20	39,420	0.29	39,543	0.17	15,705	0.23	15,770
4C	Mortality-weighted Charlson score	13	0.12	39,912	0.16	40,447	0.09	15,889	0.14	16,016

*MCS*  Short Form-8 Mental Component Score, *PCS*  Short Form-8 Physical Component Score. F-ratios for QDIS-MCC and Charlson models not reported, all *p* < .001

^a^df = degrees of freedom in the numerator

^b^Base model holdout group (female) was female, age 45–64, white, and high-school graduate

^c^These weights from developmental sample regressions at baseline were used for all Model 2 comparisons, cross-sectional and longitudinal including cross-validations

^d^Model 3 used developmental sample baseline individualized QDIS scores weights (see text). All models adjusted for Base model main effects of (# of categories): age (6), gender (2), education (4) race (4). Intercept (holdout group) was ages 18–34 years, female, with 12 years of education and white. Intercept values (constants) varied across models, from 43.8 to 52.8 for PCS and from 45.8 to 52.7 for MCS. Values were lowest for Base and highest for Model 3, respectively

Results from longitudinal comparisons of models predicting PCS and MCS are presented in the right-most columns of Table 5. Although longitudinal adjusted R^2^ estimates were typically lower than cross-sectional estimates for both PCS and MCS, the progression of R^2^ and AIC estimates across models was largely the same. As with cross-sectional tests, longitudinal predictions were stronger for PCS in comparison to MCS. To summarize, the observed pattern of results across methods supports progressive incremental validity beyond a sociodemographic-only base model of an expanded condition checklist, population QOL over mortality weighting, and individualized over population QOL weighting for purposes of estimating PCS and MCS in cross-sectional and longitudinal analyses.

Figure 1 illustrates the practical implications of differences in MCC multimorbidity and comorbidity[38, 39] aggregation methods for predictions of physical QOL (PCS) for adults with osteoarthritis (OA), the most prevalent pre-ID condition in the developmental sample (N = 1135). Predictions from these models, yielding a single effect size estimate for conditions *comorbid*, among those with OA are shown in Fig. 1 as horizontal lines because they apply the same (average) adjustment to all OA-specific strata. Lower and higher lines, respectively, indicate larger and smaller adjustments in PCS across methods. Model #4C (R^2^ = 0.162, *p* < 0.001), which applied a population mortality-weighted adjustment of −0.11 SD (t = −21.9, *p* < 0.001) shown by the green dashed line, is the smallest population-weighted adjustment. From Model #3 (R^2^ = 0.542, *p* < 0.001), the blue dotted line shows a much larger adjustment of −0.53 SD (t = −25.5, *p* < 0.001) using the individualized sum of QDIS comorbidity ratings. It is noteworthy that adjustments for the latter two models did not include OA, which is excluded from the CCI and omitted, by design, for estimating QDIS MCC comorbidity in OA analyses.

In contrast, Model #3 (R^2^ = 0.394, *p* < 0.001) adjustments are displayed in Fig. 1 as blue box plots of PCS outcomes for individuals in each OA-specific QDIS global item impact category. Increases in separations between categories in adjusted PCS scores are roughly constant at about -0.5 SD, as box plots decline with increasing QOL impact attributed to OA (from left to right). In comparisons with the None category, PCS means were lower for Little (t = −9.46, p < 0.001), Some (t = −20.82, *p* < 0.001) and Lot/Extremely (t = −30.24, *p* < 0.001) impact categories. As noted for the developmental sample analysis (Table 5), controlling for comorbidity using the QDIS method substantially increased R^2^. For OA and other pre-identified conditions, these gains in accuracy were almost entirely due to substantial reductions in score range and interquartile ranges within categories (data not reported).

In comparisons with population norms, results from individual estimates illustrated in the four blue box plots reveal systematic biases: (1) errors *under-estimated* PCS outcomes for adults attributing No or Little specific impact to OA and (2) errors *over-estimated* outcomes for individuals attributing higher levels of specific impact to OA. The percentages of over- and under-estimation errors exceeding minimally important difference (MID) criterion [67] are shown at the bottom of Fig. 1 for each OA-specific impact category, with overestimations ranging from 24 to 96% and underestimations from 20% to < 1% across impact groups.

### Cross-validation of MCC impact aggregation models

As summarized in Table 6, independent cross-validations yielded the same pattern of results as observed with the developmental sample, replicating the progressive increases in R^2^ and decreases in AIC values, as hypothesized, for both PCS and MCS scores in cross sectional and longitudinal analyses. However, some adjusted R^2^ estimates were slightly lower than observed with the developmental sample.

**Table 6:** Summary of variances explained and model fit by method and model, cross-validation sample

Model & Description		df	Cross-sectional models N = 3416				Longitudinal models N = 1153			
			MCS		PCS		MCS		PCS	
			Adj R^2^	AIC	Adj R^2^	AIC	Adj R^2^	AIC	Adj R^2^	AIC
*QDIS-MCC Models*										
0	Base model – sociodemographic variables only	12	0.06	24,926	0.09	24,512	0.05	8487	0.07	8405
1	Simple chronic conditions count	13	0.20	24,362	0.30	23,624	0.18	8317	0.24	8163
2–M/P	Population-weighted MCS/PCS score	13	0.30	23,930	0.40	23,096	0.25	8221	0.34	8000
3*	Individualized QDIS-weighted MCC score	13	0.30	23,944	0.41	23,048	0.26	8206	0.35	7998
*Charlson Models*										
1C	Charlson 12 count	13	0.12	24,703	0.21	24,024	0.12	8404	0.17	8277
2C–M/P	MCS-/PCS-weighted Charlson score	13	0.12	24,703	0.22	23,980	0.11	8407	0.18	8258
3C*	Individualized QDIS-weighted Charlson score	13	0.17	24,509	0.28	23,688	0.15	8354	0.24	8176
4C	Mortality-weighted Charlson score	13	0.10	24,764	0.18	24,152	0.09	8435	0.14	8318

*MCS*  Short Form-8 Mental Component Score, *PCS* Short Form-8 Physical Component Score, *df*  degrees of freedom in the numerator. F-ratios for QDIS-MCC and Charlson models not reported, all *p* < .001. *Uses baseline individualized QDIS scores

## Discussion

Systematic comparisons among unique features of different MCC aggregation methods, while holding other features constant, linked improved accuracy to: (a) use of an expanded list of chronic conditions, (b) population weighting of reported conditions in terms of their *QOL impact*, as opposed to *mortality* weighting, and (c) use of *individualized* estimates of QOL impact for each condition rather than its population weight that ignores individual differences within each condition. An expanded chronic condition checklist paired with *individualized* disease-specific QOL impact measures standardized across multiple chronic conditions (QDIS-MCC) enabled the largest improvements increasing the accuracy of predictions of physical and mental QOL outcomes into the moderate to strong model range [9].

Our findings have implications for purposes of group comparisons in outcomes research and improving individual patient quality of care. These include: (a) more confidently attributing QOL differences observed between self-selected groups to the effects of group membership as opposed to case-mix differences [3–8, 12–16], (b) adding better QOL estimates of individual experiences of clinical care and treatment outcomes, consistent with the principles of clinimetrics [11] to improve tailored treatment decision making [16, 18], (c) determining whether individuals are currently functioning and feeling better/worse than expected for their age, comorbid conditions, and other characteristics; and (d) identifying those more or less likely to experience clinically significant improvement as a result of treatment [15, 16, 68–70].

Figure 1 for adults with pre-identified OA illustrates how individualized disease-specific impact ratings work to improve QOL prediction accuracy and reveals systemic patterns of substantial over- or under-estimation errors from using the population main effect adjustments based on the legacy comorbidity methods studied. Applying the same population adjustment for all with a given condition tilts this teeter-totter pattern of errors up or down depending on adjustment magnitude, whereas individualized adjustments reduced both over- and under-estimation errors. Given that only errors > 0.5 SD units were counted, the large percentage of errors observed are likely of importance given that they exceed clinically, economically, and socially important effect sizes in the range of 0.2–0.3 SD units recommended by developers of the generic QOL outcome measures studied [17, 53, 54, 67]. Although adjusted R^2^ estimates were consistently lower for mental (MCS) compared to physical (PCS) predictions, improved incremental validity (or predictive validity beyond that provided by legacy methods) was consistently observed for individualized estimates in predictions of both PCS and MCS.

At the core of the improved MCC impact estimation is a psychometrically-sound summary disease-specific QOL impact score. Despite the breadth of QOL domains represented in the standardized QDIS item bank for each specific condition, those items are sufficiently homogeneous to justify a 1-factor model summary score [24, 25]. Further, the single global QDIS item representing each disease-specific bank correlates highly enough (r > 0.90) with the total item bank to justify its use in the shortest possible 2–3 min QDIS-MCC survey, combining a standardized checklist and QOL impact item attributing impact specifically to each reported condition [24]. Although the evolving applications of psychometric theory and methods [71] in parallel with clinimetric principles has not been without debate [72, 73], it is important to note that differences in their emphasis are complementary and that they share some commonalities [21, 74, 75]. For example, the current study uses incremental validity methods promoted by clinimetricians [11] and by psychometricians [56]; both of which advocate for validity testing using clinical criteria.

In support of generalizing results, significant unique MCC effects in predicting PCS and MCS, as well as patterns of larger effect sizes in the current study are concordant with results from the US Medical Outcomes Study (MOS) [54], US general population surveys [53, 76, 77], as well as studies in eight other countries [55]. For example, across common conditions, negative effects on PCS were largest for arthritis, heart, and lung conditions in the US, seven European countries and Japan. Given the consistency across samples and countries and languages, it has been suggested these estimates can be generalized as a basis for defining important effect sizes [55]. Accordingly, for standardized record based and self-reported chronic condition checklists, the population weights documented in Table 4 and elsewhere [48] are recommended for use in achieving the advantages of QOL population-weighted MCC impact scoring over simple counts or population mortality weighting without additional primary data collection.

The relatively large unique effects of OA, back problems, chronic fatigue, and fibromyalgia on PCS and depression on MCS, conditions not included in the CCI, may at least partly explain its relatively poorer performance. The pattern of higher adjusted R^2^ estimates for predictions of PCS compared to MCS based on MCC is also consistent with prior research [31, 33]. Increased variances explained by expanded condition checklists in the current study (adjusted R^2^ 0.39 and 0.34, respectively) are also consistent with prior US research (adjusted R^2^ 0.45 and 0.31) [53]. Unfortunately, prior studies in Europe and Japan did not report model R^2^ estimates [55].

There are noteworthy strengths and limitations of the current study. Data came from large, nationally representative US population samples supplemented with pre-identified chronically ill adults, which enabled both interpretations of QOL in relation to more representative national norms and the greater precision from larger supplemental samples required for within-disease MCC comparisons. The potential shortcoming of regressions overfitting data was addressed by cross-validation of model comparisons in an independent sample and data from a later time point. It is a strength of the current study that (a) cross-sectional baseline developmental sample population weights for chronic conditions used in standardizing aggregate QDIS-MCC scores were cross-validated in an independent sample and (b) longitudinal (nine-month) outcome models replicated the overall pattern of cross-sectional predictions. Reliance on the 8-item MOS survey (SF-8) [24] estimates of chronic condition effects on PCS and MCS (effect sizes and adjusted R^2^ estimates) is a potential limitation, although literature suggests otherwise [53, 78]. To address this concern, model comparisons were replicated for a random subsample who completed the full-length 36-item MOS Health Survey (SF-36) in parallel with SF-8. Overall patterns of PCS and MCS results were comparable with results from other studies using the SF-36, SF-12, and SF-8 surveys [47, 53, 78].

Two other potential limitations are that all data were self-reported and collected electronically with no clinical verification of diagnoses or condition severity, and it was assumed that participants can validly rate the QOL impact of one specific condition in the presence of MCC. Addressing the first, prior research has identified discrepancies between patient self-report and administrative data, with higher rates of disagreement for cancer or mental health diagnoses compared to diabetes [79]. However, other studies suggest that patient self-reported conditions perform equally as well in predicting QOL in comparison with comorbidity data obtained from medical records [80, 81]. To the extent reliance on self-report was a limitation, it is likely to have similarly effected all MCC aggregation methods tested. Second, QDIS global item and multi-item attributions to a specific condition have been shown to be sufficiently valid [27] in the presence of MCC. Specifically, for pairs of pre-identified and other comorbid conditions studied here (asthma, diabetes, OA), correlational tests supported convergent (same condition-different methods and criteria) and discriminant validity (different conditions, same method) in more than 90% of tests [27]. Some noted exceptions involving MCC characterized by the same symptom (e.g., SOB) warrant further study. In such cases, the individualized multimorbidity QOL impact aggregation provides a better case-mix adjustment for predicting generic QOL outcomes, in comparison with a simple count, population QOL- and mortality-weighted methods. While clinic data and judgement are still required to discern among confounded causes of patient experiences, attribution of ambiguous symptoms to a specific condition is not as informative as the extent of QOL impairment.

Finally, data limitations noted above limited method comparisons to 12 conditions common to the CCI and 35-item checklist. Further, some CCI conditions required data that were only available for the developmental sample with pre-identified conditions. While the CCI was scored conservatively, consistent with previous studies [59], excluded conditions may have contributed to relatively poorer CCI performance. However, it should be noted that the CCI is less comprehensive, omitting more than a dozen conditions (e.g., OA) shown to significantly diminish QOL [53, 76, 77]. The Elixhauser index [35, 82] is a more comprehensive alternative to the CCI, although it requires specific ICD coding (beyond 3 digits) for accuracy, still excludes conditions known to adversely impact QOL (e.g., fibromyalgia, migraines), and has limited potential to discriminate severity of impact within each condition. All comparisons of aggregation methods in terms of simple counts versus population QOL and mortality weighting, and individualized weighting in this study were standardized using the 12 common CCI conditions. The optimal number and selection of specific checklist conditions used to standardize adjustments for chronic condition case-mix differences in QOL outcomes monitoring warrants further attention.

Practical considerations present other potential limitations, particularly for individualized QDIS-MCC impact assessments that require primary data collection, which increase costs and respondent burden. Whereas use of more practical generic QOL measures is increasing in EHRs, short-form solutions have only recently been available for disease-specific measures due to length of legacy tools and lack of disease-specific QOL impact comparability across conditions [23]. Single-item QDIS measures with specific attributions to each condition reported in the current study are substantially more practical. They yield directly comparable scores that correlate highly with their full QDIS item bank for the same condition, and are valid in relation to full-length legacy measures of the same disease, despite their coarseness and lower reliability [24, 27]. Supplementing the global QDIS item used for each disease in the current study with multi-item paper–pencil or internet-based CAT administrations of items making attributions to the same disease has been shown to increase precision for clinical research and practice. [24, 26] This adaptive logic is the next step when more reliable individualized estimates (e.g., likelihood of treatment relief) are needed [16]. Feasibility, respondent burden reduction, and clinical utility of such adaptive logic were supported in a national registry pilot study before and after joint replacement, where responsiveness and high correlations between QDIS-OA, QDIS-MCC, and generic PCS outcomes were statistically significant despite a very small sample [83]. Other findings suggest there are points beyond which additional measurement precision may not be worth the burden and cost [26, 41]. Further condition-by-condition research is recommended to optimize adaptive logic for patient selection to maximize measurement efficiency. Other issues warranting further study are whether MCC aggregation methods shown to be more predictive of generic QOL are also more predictive of other outcomes (e.g., hospitalization, job loss, and costs of care). Given that simple condition counts have been shown to predict such other outcomes [8, 29], it is reasonable to hypothesize that individualized estimates will do even better.

To summarize, individualized single-item measures of QOL impact with standardized content and scoring across MCC, that differ only in attribution to a specific condition, provided a more practical method of aggregating MCC QOL impact. This new comorbidity index (QDIS-MCC) was more useful than legacy MCC aggregation methods for purposes of adjusting for case-mix differences in predicting generic physical and mental QOL outcomes. The QDIS-MCC short form combines a standardized chronic condition checklist with a single global QDIS impact item for each reported condition and required less than three minutes for most respondents to complete (median one minute for checklist, median two minutes for comorbid QDIS item administrations). This approach illustrates the potential for improving the staging of individual patients, deciding whether more reliable (e.g., additional) measurement is likely to be worthwhile, and providing a better adjustment for individual and group case-mix differences in MCC burden for purposes of more accurately predicting generic physical and mental QOL outcomes. For comparative effectiveness research, such advances can strengthen case mix adjustments essential to attributing differences in health outcomes across self-selected groups [57]. In clinical practice, individualized disease-specific MCC QOL impact stratifications can provide actionable information about the severity of MCC accounting for likely differences in patient’s generic health status and outcomes. To assure the availability of QDIS-MCC forms for further research by scholars and individuals for academic research, the non-profit MAPI Research Trust (MRT) is managing and distributing licenses for use worldwide (https://mapi-trust.org/) for a minimal handling fee. MRT is also handling commercial licenses to companies, healthcare delivery organizations, and others for commercial applications.

## Supplementary Information

**Additional file 1:** QDIS-MCC Combined Checklist and QOL Impact: Paper-pencil form.

## Data Availability

Data generated and/or analyzed during the current study are not yet available in a public depository due to ongoing peer review of study publications, but are available from the corresponding author on reasonable request.

## Abbreviations

AIC: Akaike information criterion
CCI: Charlson Comorbidity Index
CCC: Chronic condition checklist
CKD: Chronic kidney disease
CVD: Cardiovascular disease
COPD: Chronic obstructive pulmonary disease
CHF: Congestive heart failure
EDC: Electronic data collection
MI: Myocardial infarction
MCC: Multiple chronic conditions
MCS: Short Form 8 health survey, mental component summary
OA: Osteoarthritis
PCS: Short Form 8 health survey, physical component summary
PRO: Patient-reported outcome
QDIS: Quality of life disease impact scale
QDIS-MCC: Quality of life disease impact scale for multiple chronic conditions
QOL: Quality of life
RA: Rheumatoid arthritis
