# Supplementary material for: Improving multimorbidity measurement using individualized disease-specific quality of life impact assessments: predictive validity of a new comorbidity index
Source: Health Qual Life Outcomes. 2022 Jul 12;20:108. doi: 10.1186/s12955-022-02016-7 (PMC9277868; doi:10.1186/s12955-022-02016-7)
Supplement: Supplementary file 1 — Additional file 1: QDIS-MCC Combined Checklist and QOL Impact: Paper-pencil form. [file 12955_2022_2016_MOESM1_ESM.pdf]

Chronic Conditions and How They Affect You

Have you ever been told by a doctor or other health professional that you had any of the following conditions?

On the left, please answer NO or YES for each condition. If YES, on the right, please answer the question for each condition you have.

Please answer NO or YES for each condition			If YES	If YES, in the past 4 weeks, how much did this CONDITION limit your everyday activities or your quality of life?				
**CONDITION**	**NO**	**YES**		**Not at all**	**A little**	**Some**	**A lot**	**Extremely**
Hypertension, sometimes called high blood pressure	☐	☐	➡	☐	☐	☐	☐	☐
A heart attack in the last year, also called myocardial infarction	☐	☐	➡	☐	☐	☐	☐	☐
Congestive heart failure or enlarged heart	☐	☐	➡	☐	☐	☐	☐	☐
Diabetes or high blood sugar	☐	☐	➡	☐	☐	☐	☐	☐
Angina (An-JI-na or AN-ji-na)	☐	☐	➡	☐	☐	☐	☐	☐
Stroke	☐	☐	➡	☐	☐	☐	☐	☐
Cancer, except skin cancer	☐	☐	➡	☐	☐	☐	☐	☐
Asthma	☐	☐	➡	☐	☐	☐	☐	☐
Chronic Obstructive Pulmonary Disease (COPD)	☐	☐	➡	☐	☐	☐	☐	☐
Kidney Disease	☐	☐	➡	☐	☐	☐	☐	☐
Rheumatoid arthritis	☐	☐	➡	☐	☐	☐	☐	☐
Osteoarthritis, degenerative arthritis	☐	☐	➡	☐	☐	☐	☐	☐
Osteoporosis	☐	☐	➡	☐	☐	☐	☐	☐
Ulcer or stomach disease such as gastritis or duodenitis	☐	☐	➡	☐	☐	☐	☐	☐
Liver infection (Hepatitis B or C)	☐	☐	➡	☐	☐	☐	☐	☐
Irritable bowel syndrome (or functional bowel syndrome or other chronic bowel disease)	☐	☐	➡	☐	☐	☐	☐	☐
Obesity	☐	☐	➡	☐	☐	☐	☐	☐
HIV or AIDS	☐	☐	➡	☐	☐	☐	☐	☐
Anemia	☐	☐	➡	☐	☐	☐	☐	☐
Clinical depression	☐	☐	➡	☐	☐	☐	☐	☐
Chronic fatigue syndrome (CFS)	☐	☐	➡	☐	☐	☐	☐	☐
Fibromyalgia (FM)	☐	☐	➡	☐	☐	☐	☐	☐
Migraine headaches	☐	☐	➡	☐	☐	☐	☐	☐
Enlarged prostate or Benign prostate (BPH)	☐	☐	➡	☐	☐	☐	☐	☐

Quality of Life Disease Impact Scale (QDIS®) in Chronic Condition Checklist format. Copyright © 2010-2017 John Ware Research Group, Inc. All rights reserved.

Chronic Conditions and How They Affect You

Do you now have any of the following conditions?

On the left, please answer NO or YES for each condition. If YES, on the right, please answer the question for each condition you have.

Please answer NO or YES for each condition			If YES	If YES, in the past 4 weeks, how much did this CONDITION limit your everyday activities or your quality of life?				
**CONDITION**	**NO**	**YES**		**Not at all**	**A little**	**Some**	**A lot**	**Extremely**
Hypothyroidism or underactive thyroid condition	☐	☐	➡	☐	☐	☐	☐	☐
Chronic allergies or sinus trouble	☐	☐	➡	☐	☐	☐	☐	☐
Seasonal allergies, such as hay fever	☐	☐	➡	☐	☐	☐	☐	☐
Chronic back problems or sciatica	☐	☐	➡	☐	☐	☐	☐	☐
Trouble seeing, even when wearing glasses or contact lenses; blind or unable to see at all	☐	☐	➡	☐	☐	☐	☐	☐
Deafness or other trouble hearing with one or both ears	☐	☐	➡	☐	☐	☐	☐	☐
Dermatitis or other chronic skin conditions	☐	☐	➡	☐	☐	☐	☐	☐
Limitation in the use of an arm or leg (missing, paralyzed, or weakness)	☐	☐	➡	☐	☐	☐	☐	☐
Joint problems of the foot or ankle	☐	☐	➡	☐	☐	☐	☐	☐
Joint problems of the hip or knee	☐	☐	➡	☐	☐	☐	☐	☐

Quality of Life Disease Impact Scale (QDIS®) in Chronic Condition Checklist format. Copyright © 2010-2017 John Ware Research Group, Inc. All rights reserved.
